# Data on synthesis of oligomeric and polymeric poly(butylene adipate-co-butylene terephthalate) model substrates for the investigation of enzymatic hydrolysis

**DOI:** 10.1016/j.dib.2016.02.029

**Published:** 2016-02-19

**Authors:** Veronika Perz, Klaus Bleymaier, Carsten Sinkel, Ulf Kueper, Melanie Bonnekessel, Doris Ribitsch, Georg M. Guebitz

**Affiliations:** a acib – Austrian Centre of Industrial Biotechnology, Konrad Lorenz Strasse 20, 3430 Tulln, Austria; b acib – Austrian Centre of Industrial Biotechnology, Petersgasse 14, 8010 Graz, Austria; c BASF SE, Carl-Bosch-Strasse 38, 67056 Ludwigshafen, Germany; d Institute of Environmental Biotechnology, University of Natural Resources and Life Sciences, Konrad Lorenz Strasse 20, 3430 Tulln an der Donau, Austria

**Keywords:** Ada, adipic acid, Ba, benzoic acid, BaBTaBBa, bis(4-(benzoyloxy)butyl) terephthalate, BTa, mono(4-hydroxybutyl) terephthalate, BTaB, bis(4-hydroxybutyl) terephthalate, Da, decanoic acid, DaBTaBDa, bis(4-(decanoyloxy)butyl) terephthalate, H, 1,6-hexanediol, Ha, hexanoic acid, HaBTaBHa, bis(4-(hexanoyloxy)butyl) terephthalate, HTaH, bis(4-hydroxyhexyl) terephthalate, PBAT, (poly(butylene adipate-co-butylene terephthalate), Ta, terephthalic acid, Tda, tetradecanoic acid, TdaBTaBTda, bis(4-(tetradecanoyloxy)butyl) terephthalate, THF, tetrahydrofuran., Oligomer synthesis, Oligomer analysis, Polyester model substrates

## Abstract

The aliphatic-aromatic copolyester poly(butylene adipate-co-butylene terephthalate) (PBAT), also known as ecoflex, contains adipic acid, 1,4-butanediol and terephthalic acid and is proven to be compostable [1], [2], [3]). We describe here data for the synthesis and analysis of poly(butylene adipate-co-butylene terephthalate variants with different adipic acid:terephatalic acid ratios and 6 oligomeric PBAT model substrates. Data for the synthesis of the following oligomeric model substrates are described: mono(4-hydroxybutyl) terephthalate (**BTa**), bis(4-(hexanoyloxy)butyl) terephthalate (**HaBTaBHa**), bis(4-(decanoyloxy)butyl) terephthalate (**DaBTaBDa**), bis(4-(tetradecanoyloxy)butyl) terephthalate (**TdaBTaBTda**), bis(4-hydroxyhexyl) terephthalate (**HTaH**) and bis(4-(benzoyloxy)butyl) terephthalate (**BaBTaBBa**). Polymeric PBAT variants were synthesized with adipic acid:terephatalic acid ratios of 100:0, 90:10, 80:20, 70:30, 60:40 and 50:50. These polymeric and oligomeric substances were used as ecoflex model substrates in enzymatic hydrolysis experiments in the article “Substrate specificities of cutinases on aliphatic-aromatic polyesters and on their model substrates” [4].

**Specifications Table**

**Table:** 

Subject area	Chemistry
More specific subject area	Organic synthesis, synthesis of model substrates for enzymatic hydrolysis experiments and biodegradation tests
Type of data	Synthesis protocols, figure, table, text file
How data was acquired	^1^H NMR and ^13^C NMR (Varian Inova 400 and DPX 400, CDCl_3_, C_2_D_2_Cl_4_, d-DMSO, tetramethylsilane as internal standard).HPLC (Agilent Series 1100 HPLC system using a Symmetry C_18_, 5 µm, 4.6 mm×250 mm column (Waters Corporation, Milford, USA)).
Data format	Analyzed data
Experimental factors	Starting compounds were either purchased or synthesized using already published synthetic protocols
Experimental features	Model substrates were synthesized and ^1^H NMR, ^13^C NMR and HPLC verified their identity and purity.
Data source location	Ludwigshafen, Germany and Tulln an der Donau, Austria
Data accessibility	The data are supplied with this article

**Value of the data**

• The data indicate the suitability of oligomers as model substrates for bulky polymers.
• The data offer the option to investigate the influence of polymer structures and the mechanism of biodegradation.
• The data allow identification of substrate specificities of enzymes and detailed analysis of cleavage sites during polymer degradation.

## Data

Data presented here describe the additional chemical analysis of poly(butylene adipate-co-butylene terephthalate [1], [2], [3] variants and oligomeric model substrates. 1H NMR spectra and 13C NMR spectra are provided showing the chemical structure and purity ob synthesized chemicals.

## 1 Experimental design, materials and methods

### 1.1 General

**Fig. 1:**
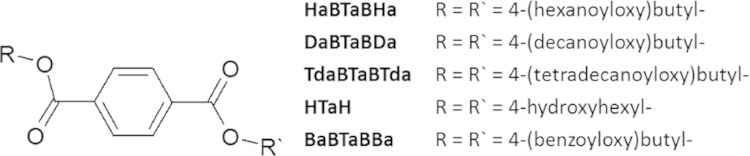
Overview of chemical structures of oligomeric substrates.

The identity and purity of the final products as well as the intermediate products were verified by ^1^H-NMR and ^13^C-NMR (Varian Inova 400 and DPX 400) using CDCl_3_, C_2_D_2_Cl_4_, d-DMSO, tetramethylsilane as internal standards. HPLC analysis was carried out on an Agilent Series 1100 HPLC system using a Symmetry C_18_, 5 µm, 4.6 mm×250 mm column (Waters Corporation, Milford, USA). The gradient was started at 90% A (0.1 vol% H_3_PO_4_ in water), 10% B (0.1 vol% H_3_PO_4_ in acetonitrile) at 10 min, followed by 50% A and 50% B at 20 min, 5% A and 95% B at 30 min, 5% A and 95% B at 32 min, 90% A and 10% B. This concentration was held until minute 40 to equilibrate the system. The flow rate was set to 1 ml min^−1^ and the temperature to 20 °C. The eluted products were detected by the UV detector at 220 nm. Chemical structures of the produced PBAT model substrates are presented in Fig. 1.

### 1.2 Synthesis of oligomeric PBAT model substrates

#### 1.2.1 Synthesis of mono(4-hydroxybutyl) terephthalate (BTa)

*Step 1* was performed according to Padias and Hall [5].

*Step 2*: Thionylchloride (980 g, 8.24 mol, 11.4 eq) was added dropwise to a mixture of monomethyl terephthalate (129.1 g, 0.72 mol, 1 eq) and 556 g of toluene. The reaction mixture was then stirred under reflux (80 °C) for 3 h and turned into a yellow solution. The solvent and excess of thionylchloride were removed under reduced pressure to give 122.6 g of methyl terephthaloyl monochloride (86% yield).

*Step 3*: 1,4-Butanediol (231 g, 2.56 mol, 8.3 eq) and pyridine (124.2 g, 1.57 mol, 5.1 eq) were cooled to 0 °C. A solution of methyl terephthaloyl monochloride (61.3 g, 0.31 mol, 1 eq) and 233 g of methylenchloride was added dropwise over 15 min and it was stirred for further 2 h at room temperature. Then, the reaction mixture was poured into 2 kg of water and brought to pH 1 using 1.5 kg of 1 M HCl. The aqueous phase was extracted using methylene chloride. The solvent was removed under reduced pressure to give 63 g of methyl hydroxybutyl terephthalate (90–95% by ^1^H NMR, 73–77% yield).

*Step 4*: Methyl hydroxybutyl terephthalate (22 g, 0.087 mol, 1 eq) was dissolved in 885 ml of tert-butyl methyl ether (MTBE) and chloroform (ratio 1:1 v/v). Dihydropyran (18.3 g, 0.22 mol, 2.5 eq) was added dropwise over 15 min and then 0.8 g of conc. HCl were added to result in a pH of 4. The reaction mixture was stirred for further 18 h at room temperature. The solution was then washed with 314 g of saturated sodium bicarbonate solution and 373 g of water. After drying and removing the solvent under reduced pressure, 38.4 g of crude product were obtained. This crude product was purified by flash chromatography (hexane:ethyl acetate 8:1). 23.8 g of the dihydropyranyl derivative of methyl hydroxybutyl terephthalate was obtained (98.6 HPLC-a%, 80% yield).

**Fig. 2:**
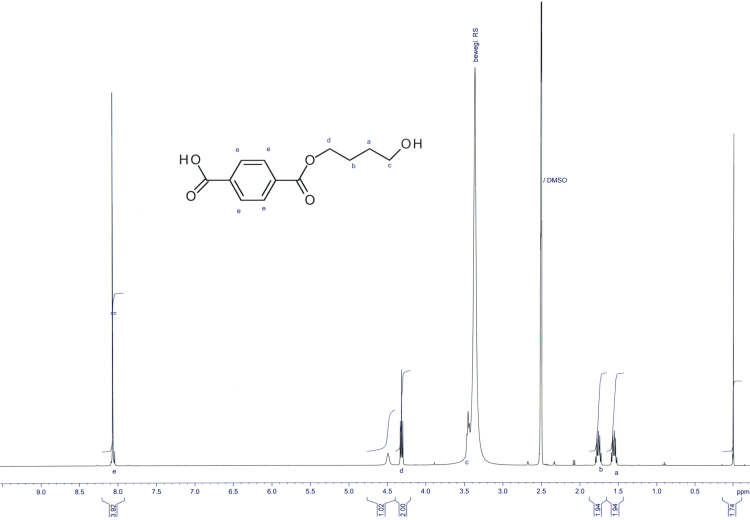
^1^H NMR spectrum of monohydroxybutyl terephthalate (**BTa**).

**Fig. 3:**
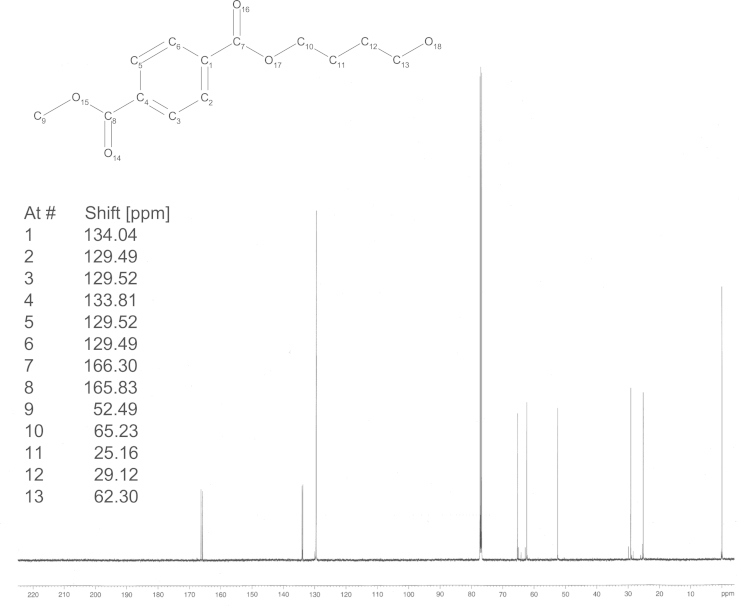
^13^C NMR spectrum of monohydroxybutyl terephthalate (**BTa**) scaffold.

*Steps 5 and 6*: The dihydropyranyl derivative of methyl hydroxybutyl terephthalate (5.08 g, 98.6 HPLC-a%, 16 mmol, 1 eq) and DABCO (2.67 g, 98%, 23 mmol, 1.5 eq) are sealed in a tube and heated to 100 °C for 4 h. At room temperature, the oil was taken up in 100 ml water. To this opaque solution, 25 ml of 10% H_2_SO_4_ were added. The reaction mixture was heated to 90 °C and then diluted with 75 ml of water to obtain a clear solution. Then, the reaction mixture was allowed to cool to room temperature and was stirred for further 2 h. The obtained precipitate was filtered off, washed two times with 20 ml of water each and finally dried in a nitrogen stream. 2.92 g of monohydroxybutyl terephthalate were obtained. Recrystallization from toluene resulted in 2.42 g of monohydroxybutyl terephthalate (96.6 wt% by ^1^H NMR, 63% yield; Figs. 2 and 3).

#### 1.2.2 Synthesis of the BTaB derivates HaBTaBHa, DaBTaBDa, TdaBTaBTda, HTaH and BaBTaBBa

**Fig. 4:**
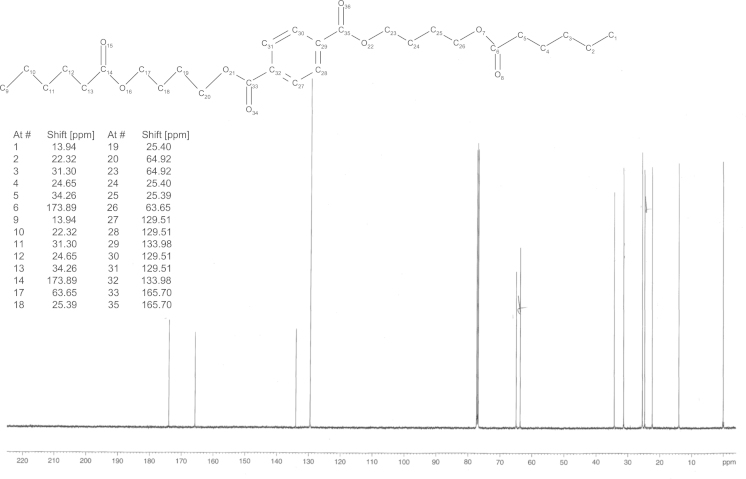
^13^C NMR spectrum of bis(4-(hexanoyloxy)butyl) terephthalate (**HaBTaBHa**).

**Fig. 5:**
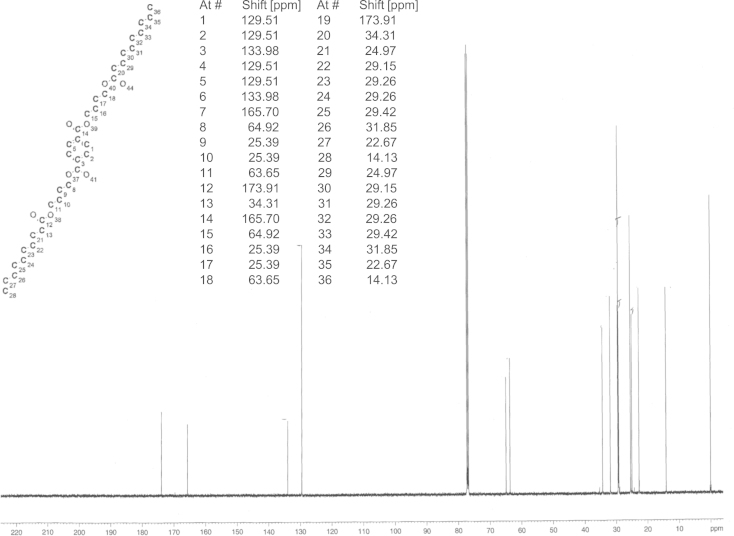
^13^C NMR spectrum of bis(4-(decanoyloxy)butyl) terephthalate (**DaBTaBDa**).

**Fig. 6:**
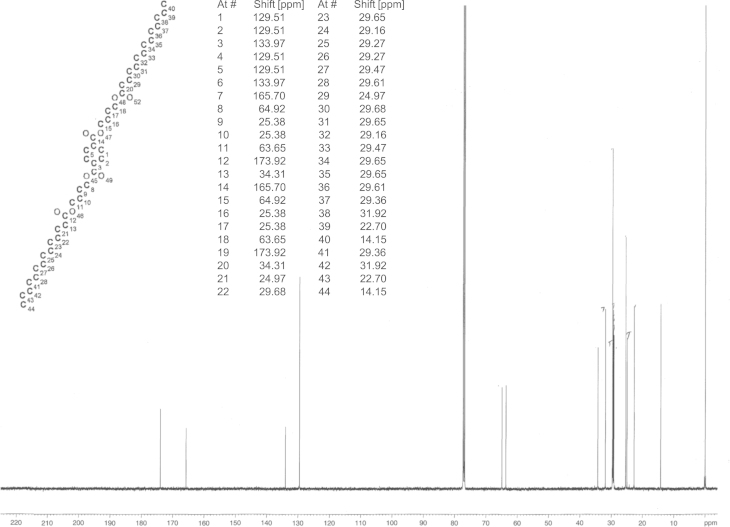
^13^C NMR spectrum of bis(4-(decanoyloxy)butyl) terephthalate (**TdaBTaBTda**).

For the synthesis of **HaBTaBHa**, 5 g **BTaB** (16 mmol, 1 eq, 91.9 HPLC-a%), 3.15 g triethylamine (32 mmol, 2 eq) and 60 ml toluene were stirred at room temperature while 4.35 g hexanoyl chloride (32 mmol, 2 eq) were added dropwise. (The synthesis of **BTaB** was conducted following Hässlin et al. [6] with modifications that were previously described [4].) After 3 h of stirring at room temperature the solvent was removed under reduced pressure and the crude product was purified by means of flash chromatography (hexane:ethyl acetate 4:1). 5.4 g of **HaBTaBHa** were obtained (92.3 HPLC-a%,>95% by ^1^H NMR, 66% yield). ^13^C spectrum is shown in Fig. 3. The synthesis of **DaBTaBDa** and **TdaBTaBTda** was performed as described alike with the difference that in the case of **DaBTaBDa**, 6.1 g of decanoyl chloride (32 mmol, 2 eq) were added dropwise and during the synthesis of **TdaBTaBTda**, 7.9 g of myristoyl choride (32 mmol, 2 eq) were added instead of hexanoyl chloride. These syntheses yielded 5.9 g **DaBTaBDa** (98 HPLC-a%, 95% by ^1^H NMR, 63% yield) and 6.3 g **TdaBTaBTda** (95.9 HPLC-a%, 95% by ^1^H NMR, 56% yield). ^13^C spectra are presented in Fig. 4, Fig. 5, Fig. 6.

**HTaH** was synthesized according to Hässlin et al. [6] with some modifications. A yellow suspension of terephthalic acid dichloride (123 g, 0.6 mol, 1 eq), pyridine (71.2 g, 0.9 mol, 1.5 eq), and 200 ml of tetrahydrofuran (THF) was stirred. A colorless solution of 1,6-hexanediol (709.2 g, 6 mol, 10 eq) and 400 ml of THF were added dropwise over 45 min. Meanwhile, the reaction mixture was slowly heated. After addition was complete, the reaction mixture was stirred for further 3 h at reflux temperature (78 °C). Then, at normal pressure, 163 g of THF were distilled off. The residue was brought to room temperature and poured into 2 l ice water. After standing over night, the precipitate was filtered off. The precipitate was taken up with 1.5 l of distilled water and stirred at 50 °C. The mixture was filtered off and the residue was taken up in 1000 ml ethanol at room temperature and filtered off after 2 h stirring. The obtained filtrate was brought to 0 °C. The obtained crystals were filtered off and dried. 54.6 g of **HTaH** were obtained (94 HPLC-a%, 23% yield, byproduct: **HTaHTaH**).

**BaBTaBBa** was prepared by stirring 140.7 g of 4-chlorobutanol (1.1 mol, 1 eq) and 155 g benzoyl chloride (1.1 mol, 1 eq) at 110 °C for 24 h. A suspension of 91.6 g terephthalic acid (0.55 mol, 0.5 eq) and 161 g DMF (2.2 mol, 2 eq) was added dropwise to the benzoyl protected butyl chloride and subsequently 223.1 g triethylamine was added dropwise (2.2 mol, 2 eq). The reaction mixture was stirred for 24 h at reflux and then extracted using 1350 g toluene. The solid was removed through filtration. The organic phase was washed with 450 g of saturated sodium chloride solution and 500 g of distilled water. Finally, anhydrous magnesium sulfate was used for drying the organic phase and the solvent was removed under reduced pressure. Crude **BaBTaBBa** was purified by means of flash chromatography (hexane:ethyl acetate 4:1) and 132 g of product were received (98.3 wt% by quant. ^1^H-NMR, 46% yield).

### 1.3 Synthesis of polymeric PBAT model substrates

#### 1.3.1 Synthesis of polybutyleneadipate

For the synthesis of **polybutyleneadipate**, 876.84 g of adipic acid (6 mol, 1 eq), 702.9 g of 1,4-butanediol (7.80 mol, 1.3 eq) and 2.56 g of tetra-n-butyl orthotitanate (7.52 mmol) were stirred at 160 °C with a slight stream of nitrogen applied. Water was distilled from the reaction mixture. The temperature was then slowly increased to 200 °C for 15 min to complete the esterification. To build up molecular weight the temperature was first reduced to 165 °C and high vacuum was applied. Temperature was then increased slowly to a maximum of 230 °C and butanediol distilled from the reaction mixture. After 6 h under high vacuum the highly viscous melt was poured from the reaction vessel and collected on a Teflon film.

#### 1.3.2 Synthesis of poly(butylene adipate-co-butylene terephthalate)s

Exemplarily for **poly(butylene adipate-co-butylene terephthalate)s** with variable Ada:Ta ratios:

For the synthesis of **poly(butylene adipate-co-butylene terephthalate) [90:10]** 99.03 g of dimethylterephthalate (0.51 mol, 1 eq), 597.5 g of 1,4-butanediol (6.63 mol, 13 eq) and 1.1 g of tetra-n-butyl orthotitanate (3.23 mmol) were stirred at 190 °C. A slight stream of nitrogen was applied. Methanol was distilled from the reaction mixture. As soon as methanol distillation was completed, 670.78 g of adipic acid (4.59 mol, 9 eq) were added to the reaction mixture. On addition, the reaction temperature dropped significantly. To remove the water—formed by esterification of adipic acid—from the reaction mixture, the temperature was raised again to 200 °C. After the water distillation was finished, an additional 1.1 g of tetra-n-butyl orthotitanate (3.23 mmol) was added to the melt and high vacuum was applied to distill butanediol from the mixture. The reaction temperature was slowly increased to 220 °C. After 3 h at this temperature, a highly viscous melt was poured from the reaction vessel and collected on a Teflon film. Ada:Ta ratios were determined using ^1^H NMR spectroscopy (Table 1).

## Figures and Tables

**Table 1:** Poly(butylene adipate-co-butylene terephthalate) substrates and their respective adipic acid to terephthalic acid ratios.

**Poly(butylene adipate-co-butylene terephthalate) type**	**Ada:Ta ratio**
Ada90_Ta10	89.3:10.7
Ada80_Ta20	78.9:21.1
Ada70_Ta30	68.9:31:1
Ada60_Ta40	58.5:41.5
Ada50_Ta50	48.8:51.2
